# Supplementary material for: Characterization of Skin Interfollicular Stem Cells and Early Transit Amplifying Cells during the Transition from Infants to Young Children
Source: Int J Mol Sci. 2024 May 22;25(11):5635. doi: 10.3390/ijms25115635 (PMC11171949; doi:10.3390/ijms25115635)

SUPPORTING INFORMATION

Supplementary Table S1: Infants (group 0-2 years) and young children (group 3-6 years) enrolled in the study.

Group	Donors	Age (years)	qRT-PCR	IF cells	RHE	Skin equivalent	MTT assay	CFE	WB
0-2 years	SK005	0,17	✓	✓					
	SK007	0,25	✓	✓	✓				
	SK0118	0,83			✓				
	SK200	1	✓	✓					
	SK004	1				✓			
	SK0148	1					✓		
	SK0144	1			✓			✓	
	SK0130	1,5					✓	✓	✓
	SK0063	1,5					✓	✓	
	SK0199	1,5	✓	✓					
	SK0023	1,5					✓	✓	
	SK195	2					✓	✓	✓
	SK010	2					✓	✓	✓
	SK0181	2					✓	✓	
	SK193	2					✓	✓	
	SK0021	2				✓			
	SK0061	2				✓			
	SK050	2				✓			
	**mean age (years)**		**0,73**	**0,73**	**0,69**	**1,75**	**1,69**	**1,69**	**1,83**
	**mean age (months)**		**8,76**	**8,76**	**8,32**	**21**	**20,25**	**20,25**	**22**
3-6 years	SK015	3					✓	✓	
	SK059	3					✓	✓	
	SK043	3					✓	✓	
	SK061	3	✓	✓					
	SK006	3		✓	✓				
	SK0201	3				✓			
	SK0130	3	✓						
	SK014	4		✓					
	SK011	5	✓	✓					
	SK009	5				✓			✓
	SK011	5					✓		
	SK002	5			✓			✓	
	SK008	6						✓	✓
	SK038	6					✓	✓	
	SK042	6	✓		✓				
	SK001	6				✓			✓
	**mean age (years)**		**4,25**	**3,75**	**4,67**	**4,67**	**4,00**	**4,33**	**5,67**
	**mean age (months)**		**51**	**45**	**56**	**56**	**48**	**52**	**68**

<u>Abbreviations:</u> IF: immunofluorescence; RHE: Reconstructed human epidermis; CFE: colony forming efficiency; WB: Western blotting.

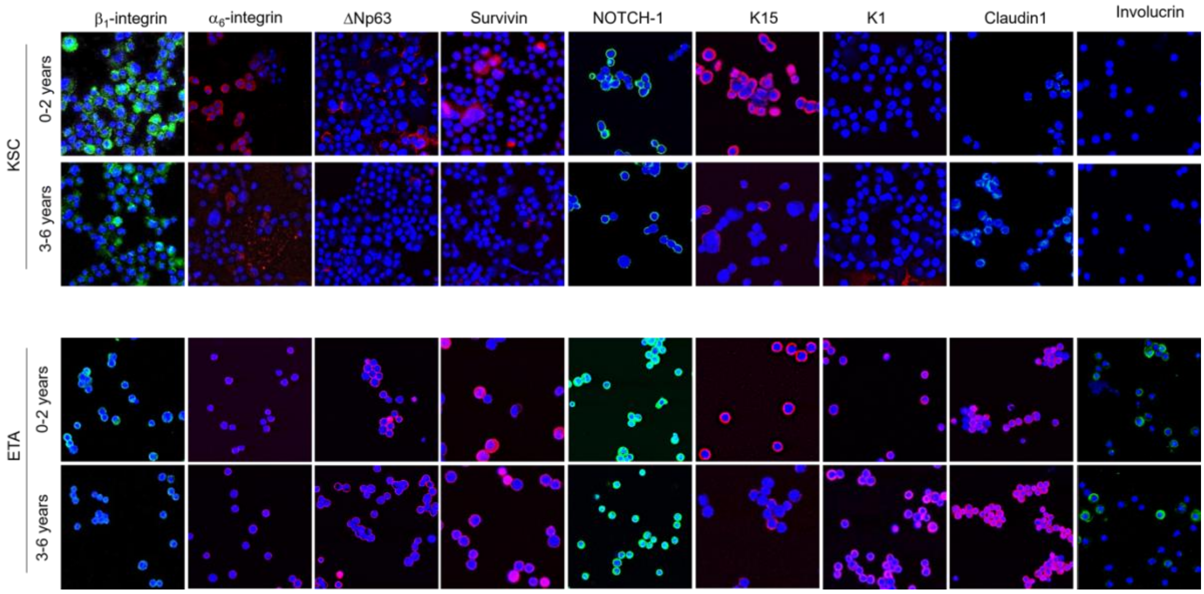

Figure S1: KSC and ETA freshly isolated cells from young children (3-6 years old) or infants (0-2 years old) were fixed and spotted onto glass slides, stained with primary antibody, and labeled with respectively anti-mouse 488 or anti-rabbit 546 Alexa Flour secondary antibodies. Nuclei were stained with DAPI. Images were recorded using a confocal scanning laser microscope.

Original Western Blotting_Fig 1

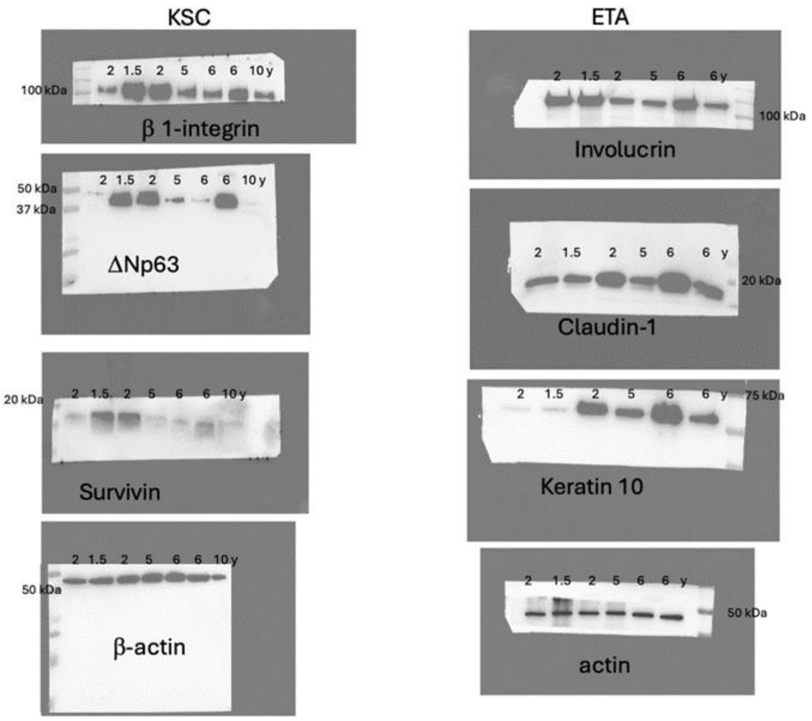

Supplement: Supplementary file 1 [file ijms-25-05635-s001.zip › ijms-2957231-supplementary.pdf]
